# Revealing study and breeding implications for production traits and tail characteristics in Simmental cattle by GWAS

**DOI:** 10.3389/fgene.2025.1491816

**Published:** 2025-01-31

**Authors:** Jie Wang, Na Shen, Kaisen Zhao, Jiayu Liao, Genglong Jiang, Jianghai Xiao, Xianbo Jia, Wenqiang Sun, Songjia Lai

**Affiliations:** ^1^ Farm Animal Genetic Resources Exploration and Innovation Key Laboratory of Sichuan Province, Sichuan Agricultural University, Chengdu, Sichuan, China; ^2^ College of Animal Science and Technology, Sichuan Agricultural University, Chengdu, China

**Keywords:** genetic analysis, milk production, meat production, phenotypic traits, tail length

## Abstract

Simmental cattle are renowned for their dual purpose as meat and dairy breeds. The study recorded phenotype data from 183 Simmental cattle and performed a Genome-Wide Association Study (GWAS) analysis to elucidate the genetic mechanisms underlying milk production, body size traits, and tail characteristics. Statistical analysis of phenotype data showed that season, parity, and age at first calving (AFC) factors had a significant effect on milk production (*P* < 0.05). The results of GWAS on cattle linear traits revealed that the candidate genes *SH3RF2, DCHS2, ADAMTS1, CAMK4, PPARGC1A, PRL, PRP6*, and *CORIN* have been found to affect body circumference (BC) and cannon circumference (CC). Through GWAS analysis of tail traits, including Circumference over tail root (COTR) and Tail Length (TL) in Simmental cattle, candidate genes associated with tail length, such as *KIF26B, ITPR2, SLC8A1*, and *SLIT3* were identified. Interestingly, candidate genes *IL1RAP, AQP9, ITPR2,* and *PKD2* were also associated with metabolic inflammation in cattle tails. These genetic markers offer valuable insights into the traits of Simmental cattle, facilitating the development of molecular breeding strategies to enhance production value and provide references for breeding programs.

## 1 Introduction

Simmental cattle hold a significant position in beef and dairy markets due to their excellent milk production performance and carcass quality (Xu et al., 2021). In addition, Simmental cattle and other dual-purpose breeds exhibit longer lifespans compared to specialized dairy breeds like Holstein, attributed to their greater rusticity and improved resilience to diseases, disorders, and stressors (Buonaiuto et al., 2024). Despite certain improvements in the current production level of Simmental cattle, the breeding program is relatively new and not yet well-established, leading to inconsistent production levels, unstable population structure, and a significant impact on the breeding and production efficiency of beef Simmental cattle. Therefore, implementing molecular breeding for the genetic improvement of the breed holds significant importance.

In practical animal husbandry, the tail plays a key role in regulating physiological activity in cattle. The tail of young calves facilitates heat dissipation through vasodilation and elevation, effectively dispersing excess heat away from the body. During periods of heat stress, other components of the circulatory system might also contribute to reducing heat load, evidenced by vasoconstriction and closer proximity of the tail to the body to conserve heat (Whittow, 1962; Walls and Jacobson, 1970). Behavioral observations shed light on various aspects of tail usage in cattle. Instances such as tail tucking towards the hind limbs are noted during instances of fright or submission, while elevated tail posture is characteristic of cows in estrus (Kiley-Worthington, 1976). Moreover, during calving, there is a significant increase in tail flick frequency and elevation, which coincides with reduced feeding behavior and rumination time (Giaretta et al., 2021). We can utilize precision livestock farming tools along with this characteristic to monitor and predict calving events in Simmental cattle in practical production settings, thereby improving management practices through timely intervention. Despite the critical role of the tail in cattle production and physiological functioning, genetic studies about this aspect remain relatively scarce, underscoring the need for further research and exploration in this field. During the investigation of the body weight and physique of Simmental cattle, a unique pattern was identified, differing from those observed in other cattle breeds: variations in tail length and circumference corresponding to different body weights. This intriguing discovery prompts us to explore the potential relationship between tail characteristics, production performance, and metabolic functions. Previous research has emphasized the genetic basis of various production traits. Based on this, the existence of genetic factors influencing body weight and metabolic-related factors in the tail characteristics of Simmental cattle is hypothesized, an area that has remained unexplored to date. Therefore, a comprehensive sequencing analysis of tail characteristics was conducted for the first time through genome-wide association studies (GWAS) to uncover genetic loci or candidate mutations affecting tail length and circumference. Additionally, GWAS analyses were performed on body circumference (BC) and cannon circumference (CC), as studies have shown that measuring BC and CC is more convenient and practical than directly measuring body weight when assessing the body condition of cattle (Udoh et al., 2021; Bima Prakasa et al., 2022). Furthermore, these supplementary physique features provide a more comprehensive and multidimensional understanding of the physique characteristics of Simmental cattle. Through the analysis of these additional traits, the aim is to discover more genetic factors related to body weight and meat production rate, thereby further enriching the understanding of the genetic mechanisms of this breed and providing additional insights into breeding strategies.

GWAS is an experimental design used to explore the correlation between genetic variations and population sample traits. This method has been successfully applied in the research fields of molecular traits such as gene expression, DNA methylation, and metabolites, promoting advances in population genetics, complex trait genetics, genetic diseases, and new therapies for diseases. S Pegolo et al. conducted GWAS analysis on slaughter and meat quality traits of 1,166 double-muscled Piemontese cattle, identifying 37 SNPs associated with 12 traits (Pegolo et al., 2020). Zhuang et al. highlighted genes with potential functions in muscle development and cell growth, such as *SQOR, TBCB, MYH10, BYADG*, and *ARHGAP31*, as candidate genes for growth traits in Simmental cattle (Zhuang et al., 2020). One of the main challenges in previous GWAS analyses was managing false positives and negatives that could occur due to population structure and familial relationships (Adhikari et al., 2023). To address this issue, a mixed linear model (MLM) combined with covariates of structure and kinship was used to control false positives.

The study aims to fill the gap in understanding the genetic basis of tail characteristics in Simmental cattle and its implications for production performance. Through comprehensive sequencing analysis and GWAS technology, the genetic loci or candidate mutations associated with tail length, circumference, body weight, and metabolic-related factors are investigated. By exploring these relationships, a deeper understanding of the genetic mechanisms underlying production metabolism and reproductive performance in Simmental cattle is achieved, while valuable molecular markers for breed identification and selection are provided. Understanding the genetic basis of tail characteristics could lead to more informed breeding strategies aimed at improving production efficiency, animal welfare, and overall profitability in the Simmental cattle industry. Additionally, the molecular markers identified in this study could serve as valuable tools for breeders in selecting animals with desired tail characteristics.

## 2 Materials and methods

### 2.1 Ethics approval

All experiments involving animals were performed under the direction of the Institutional Animal Care and Use Committee from the College of Animal Science and Technology, Sichuan Agricultural University, China (Certification No. SYXK2019-187). All methods were carried out in accordance with the relevant guidelines and regulations.

### 2.2 Animals

The animals used in this experiment were Simmental cattle, and all experimental animals used in this experiment were from the Yangping Seed Bull Farm in Meishan City, Sichuan Province, China. The reference population comprised 183 healthy cattle with complete record files, all aged 30 months or older. The cattle were housed in a well-ventilated barn with adequate space, maintained at a comfortable temperature and humidity level. The animals were provided with a balanced diet, including hay, silage, and commercial concentrate feed, which met their nutritional requirements. Fresh water was available *ad libitum*. The bedding was regularly changed to maintain hygiene, and the animals were monitored daily for signs of stress or illness. Data related to milk components were determined by an automated milk component analyzer and recorded by the ranch caretakers. And 115 individuals from this reference population were selected for further GWAS analysis.

The data related to the milk components in this study were collected using CombiFoss FT-120 Milk Composition Analyzer (FOSS, Denmark). Phenotypic data were obtained by measuring the morphological traits of Chinese Simmental cattle using measuring sticks, tape measures, calipers, and vernier calipers, by the Technical Specifications for Beef Cattle Production Performance Measurement (NY/T 2660-2014) and the Technical Regulations for Holstein Cattle Morphological Identification in China (GB/T 35568-2017). Refer to Table 1 for detailed methodology. Phenotypic data related to body size parameters, anal–genital distance, and linear evaluation indexes were recorded for all Simmental cattle. Furthermore, phenotypic data related to milk production performance, such as milk yield and milk composition, were collected for each calving. All phenotypic data were analyzed using one-way ANOVA.

**TABLE 1:** Methods for measuring various phenotypic traits.

Traits	Method of measurement	Tools
Withers Height	Vertical height from the highest point of the withers to the ground	Measuring stick
Chest Girth	Circumference around the shoulder blades	Tape measure
Body Weight	Circumference around the waist at the rear	Tape measure
Cannon Circumference	Circumference at the narrowest point of the left front leg	Tape measure
Anogenital Distance	Distance from the center of the anus to the base of the clitoris	Vernier caliper
Hip Height	Distance from the front edge of the hip bone to the rear edge of the ischium	Measuring stick
Hip Width	Distance between the two ischial tuberosities	Measuring stick
Rear Udder Height	Distance from the upper edge of the udder tissue to the base of the vulva	Tape measure
Rear Udder Depth	Distance from the rear bottom of the udder to the hock joint	Tape measure
Rear Udder Width	Distance between the attachment points on both sides of the rear udder	Tape measure
Nipple Length	Distance from the base to the tip of the nipple	Vernier caliper
Nipple Length Tail Length	The distance from the bottom of the tail to the tip	Tape measure
Circumference of Tail Root	The length of the tail at a distance of about 5 cm from the root attachment point	Tape measure

### 2.3 Genomic DNA extraction and sequencing

The cattle were restrained using a fixed neck brace in the cattle shed to ensure their safety and that of the handlers during blood collection. A 5 mL sample of whole blood was collected from each individual through the jugular vein using EDTA-containing frozen tubes. No analgesia or anesthesia was used during the blood sampling, as this procedure is minimally invasive, causes only brief discomfort, and does not justify the use of additional interventions. Following blood collection, the cattle were returned to their regular housing and continued to be raised under standard care conditions. No animals were euthanized as part of this experiment. The samples were stored at −80°C for subsequent extraction of genomic DNA. Genomic DNA was extracted from blood samples using the TIANamp Blood DNA Kit (Tiangen Biotech Company Limited, Beijing, China). DNAs with an A260/280 ratio ranging between 1.8 and 2.0 were selected for further analysis. The *Bos Taurus* genome (assembly ARS-UCD1.3) was chosen as the reference genome for predicting restriction enzyme digestion patterns based on the genome size and GC content of Simmental cattle. Subsequently, adapters with barcodes were added to each sample, followed by amplification and pooling of the samples to select the required fragments for library construction. After library construction, preliminary quantification was conducted using the Qubit 2.0 system, and the library was diluted to a concentration of 1 ng/μL. The insert size of the library was determined using the Agilent 2,100 system to ensure it fell within the expected range. Once the insert size requirements were met, the effective concentration of the library (>2 nM) was accurately quantified using qPCR. Upon successful library inspection, it used the Illumina HiSeq PE150 platform (Illumina Inc., San Diego, CA, United States).

### 2.4 Sequencing data statistics and quality assessment

After the raw image data from sequencing is transformed into sequence data through base calling, it is referred to as raw data or raw reads, and stored in the fastq file format. However, before conducting data analysis, it is necessary to remove unwanted elements such as low-quality bases and undetermined bases (represented by N) to obtain clean reads. The following methods were employed for filtering the raw data: Firstly, reads containing adapter sequences need to be filtered out. Then, paired reads should be discarded if the proportion of N bases exceeds 10% of the read length in single-end sequencing. Finally, paired reads should be removed if the proportion of low-quality bases ( ≤5) exceeds 50% of the read length in single-end sequencing. By applying strict filtering criteria to the sequencing data, high-quality clean data is obtained.

### 2.5 Single nucleotide polymorphism (SNP) detection and annotation

The high-quality data obtained after quality control was aligned to the reference genome of cattle using the BWA software (Parameters: mem -t 10 -k 32 -M) (Li and Godzik, 2006). Statistical analysis determined the alignment rate and sequencing depth per sample. SAMTOOLS software (Li et al., 2009) was used for population-level SNP detection, inferring genotype likelihood by read count at each genomic position, and applying Bayesian methods to compute allele frequencies. SNPs were filtered based on specific criteria (depth 4, missing rate ≤0.4, minor allele frequency >0.01), resulting in a high-quality SNP dataset. ANNOVAR software (version:2013-05–20) was used for SNP annotation, with SNPs classified into exonic, intronic, splice site, upstream/downstream, and intergenic regions. Coding-exon SNPs were categorized as synonymous or non-synonymous, and stop-gain/loss mutations were included.

### 2.6 Population genetic stratification assessment

The population genetic structure and phylogenetic information of the Simmental cattle natural population were analyzed using admixture software. Genetic clustering was performed assuming a range of hypothetical genetic clusters from K = 2 to K = 8, with cross-validation error (CV error) calculated for each value of K. The optimal number of clusters was determined as the K value with the lowest CV error. The resulting population structure was visualized using R software to generate a stacked bar plot of the optimal number of clusters. In addition, principal component analysis (PCA) was employed to assist in population genetic structure analysis based on SNP differences among Simmental cattle individuals. GCTA software (version 1.24.2, http://cnsgenomics.com/software/gcta/pca.html) was used to calculate feature vectors and eigenvalues, and R software was used to create a PCA distribution plot. PCA analysis was conducted as follows:

$$d_{ik}^{\prime }=\left(d_{ik}-E\left({d\;}_{\left(k\right)}\right.\right)\sqrt{E\left(d_{\left(k\right)}\right)\times \left(1-\frac{E\left(d_{\left(k\right)}\right)}{2}\right)/2}$$

In the method, $d_{ik}$ represents the SNP at position k for individual i. If individual i is homozygous for the reference allele, then $d_{ik}$ = 0; if heterozygous, then $d_{ik}$ = 1; if homozygous for the non-reference allele, then $d_{ik}$ = 2. The formula $E\left(d_{\left(k\right)}\right)$ represents the average value of $d_{k}$ . The covariance matrix of the n×n sample was calculated as X = MMT/S.

### 2.7 GWAS analysis

To enhance the accuracy of our GWAS analysis, we applied several steps in quality control and phenotypic data correction. A mixed linear model was used to adjust for fixed effects such as age, parity, season, and management practices, all of which help to isolate genetic effects. The environmental factors included average temperature and humidity for different seasons, while the management practices included feeding regimes for different lactation periods. PCA was performed to account for population stratification and relatedness, with the first few principal components included as covariates in the GWAS model. These measures ensured the phenotypic data used in the GWAS analysis were free from major confounding influences, allowing for more accurate genetic association identification. Within the scope of the study, a total of 257,436 SNPs were utilized for conducting GWAS analysis on four traits. These SNPs were obtained from an association panel comprising 115 samples. The SNPs were filtered based on specific criteria: minor allele frequency greater than or equal to 0.01, missing rate less than or equal to 0.4, and a minimum depth of 4. The GWAS was performed using the GEMMA software package (http://www.xzlab.org/software.html), which employs a mixed linear model (MLM). The mixed linear model assumed the following mode.

y=Xα+Zβ+Wμ+e

In the data analysis of the study, y represents the phenotypic trait, X is the design matrix for fixed effects (such as sex, age at calving, season, and parity, etc., with α the estimated parameter), Z is the design matrix for SNP effects (β the corresponding effects), and W is the design matrix for random effects (μ the predicted random individual). Residuals e follow a random distribution with mean zero and variance δe2. We adopted a secondary significance threshold of P < 1e-5 to address multiple hypothesis testing. This threshold was chosen to ensure robustness in identifying significant loci while accounting for the number of tested SNPs.

### 2.8 Gene functional annotation in trait-associated regions

The GALLO R software package was utilized to detect genes within a 100 kb range around significant SNP loci in the bovine genome ARS-UCD 1.3 (https://www.ncbi.nlm.nih.gov/datasets/genome/GCF_002263795.2/). The identified candidate genes were functionally annotated, and their Gene Ontology (GO) and Kyoto Encyclopedia of Genes and Genomes (KEGG) enrichment analyses were performed using the David online platform (https://david.ncifcrf.gov/) and the KOBAS database (Bu et al., 2021) with default parameters and multiple test correction. The significance threshold was set at 0.05.

## 3 Results

### 3.1 Descriptive statistics related to milk quality

#### 3.1.1 Effect of parity on milk quality

Descriptive statistics of the observed phenotypes are presented in Table 2. The effect of different parity on the average daily milk yield of Simmental cattle reached a significant level (*P* < 0.05). Simmental cattle’s average daily milk yield increased gradually with parity and decreased after reaching a peak in the third parity. Regarding the milk composition index, the milk fat percentage increased gradually with increasing parity, peaked at the fourth parity, and stabilized after that, indicating an increase in milk fat content with increasing parity. On the other hand, milk protein percentage remained relatively stable with no significant fluctuation between different parity. In addition, the number of days in lactation peaked at the third parity and then decreased with increasing parity. Table 3 presents the phenotypic data recorded for all Simmental cattle, encompassing body size parameters, anal-genital distance, and linear evaluation indexes. Furthermore, analysis in Table 3 revealed that udder development was optimal and had the largest volume in the third parity. The findings also indicated a significant impact of parity on udder depth and teat length (*P* < 0.05).

**TABLE 2:** Effect of parity on milk production performance of Simmental cattle.

Category	Parity				
	1	2	3	4	>4
Average Daily Milk Yield (kg)	18.43 ± 3.93^ab^	19.17 ± 3.20^ab^	19.30 ± 3.36^a^	18.41 ± 2.92^ab^	17.20 ± 2.67^b^
Milk Fat Content (%)	3.95 ± 0.08	3.96 ± 0.08	3.97 ± 0.06	3.98 ± 0.06	3.98 ± 0.06
Milk Protein Content (%)	3.36 ± 0.04	3.36 ± 0.05	3.37 ± 0.04	3.37 ± 0.04	3.37 ± 0.03
Lactation Days (d)	321.74 ± 93.15	331.26 ± 82.35	335.64 ± 74.01	328.01 ± 63.04	320.96 ± 70.16

^*^ The values represent the mean ± standard deviation (SD), the meaning of the symbol is: annotation, explanation of the meanings of the values in the table. ^a,b^ Values with different lowercase letters within the same row indicate significant differences (*P* < 0.05).

**TABLE 3:** Effect of parity on linear evaluation index of Simmental cattle.

Category	Parity					
	1	2	3	4	5	≥6
Hip Height (cm)	140.19 ± 6.38	140.45 ± 4.74	140.98 ± 5.26	141.25 ± 4.78	142.29 ± 4.34	139.38 ± 5.89
Hip Width (cm)	25.22 ± 4.73	25.58 ± 3.26	25.77 ± 4.99	26.37 ± 4.66	26.46 ± 4.13	28.65 ± 4.81
Angularity (mm)	32.04 ± 6.94	33.91 ± 7.12	35.87 ± 11.09	39.89 ± 12.07	35.31 ± 11.43	34.50 ± 10.40
Rear Udder Height (cm)	35.53 ± 8.73	33.14 ± 10.59	30.10 ± 9.20	31.15 ± 9.16	32.03 ± 11.38	36.00 ± 7.73
Rear Udder Width (cm)	10.43 ± 2.90	10.77 ± 2.24	12.17 ± 4.24	12.00 ± 1.86	11.65 ± 2.40	11.12 ± 4.89
Rear Udder Depth (cm)	9.94 ± 3.81^a^	7.55 ± 5.66^ab^	4.81 ± 7.69^bc^	3.88 ± 8.59^bcd^	−0.29 ± 13.41^cd^	−2.33 ± 8.94^d^
Teat Length (cm)	4.22 ± 1.11^b^	4.49 ± 0.90^ab^	4.75 ± 1.07^ab^	5.23 ± 1.01^a^	5.36 ± 1.24^a^	5.38 ± 0.86^a^
Anogenital Distance (mm)	41.38 ± 10.42	42.64 ± 7.5	44.40 ± 11.490	45.97 ± 9.39	46.97 ± 13.36	47.29 ± 10.91

^*^ The values represent the mean ± standard deviation (SD), the meaning of the symbol is: annotation, explanation of the meanings of the values in the table. ^a, b, c, d^ Values with different lowercase letters within the same row indicate significant differences (*P* < 0.05).

#### 3.1.2 Effect of age at first calving (AFC) on milk quality

Based on the statistical data in Table 4, the following conclusions could be drawn: The calving age of Simmental cattle has a certain impact on their milk production characteristics. Firstly, most Simmental cattle start calving at the age of three, indicating a general characteristic of the breed in terms of development and reproduction. Secondly, the AFC significantly affects the milk fat percentage (*P* < 0.05), with a decreasing trend as the AFC increases, suggesting that cows calving at a later age tend to produce dairy products with lower fat content. Additionally, the average daily milk yield is highest at 3 years old, followed by 4 years old, and lowest at 2 years old, indicating the influence of different AFC on milk production. However, the milk protein content tends to stabilize across different AFC, while the lactation days show an increasing trend with AFC, although not statistically significant.

**TABLE 4:** Effect of calving age on milk production characteristics in Simmental cattle.

Calving age (Months)	Average daily milk yield (kg)	Milk fat content (%)	Milk protein content (%)	Lactation days (d)
24 (n = 44)	17.71 ± 2.88	3.98 ± 0.08^a^	3.36 ± 0.04	329.23 ± 72.67
36 (n = 159)	18.51 ± 4.15	3.94 ± 0.08^b^	3.36 ± 0.04	332.48 ± 85.05
48 (n = 9)	18.36 ± 2.99	3.91 ± 0.08^ab^	3.34 ± 0.04	386.78 ± 133.23

^*^ The values represent the mean ± standard deviation (SD), the meaning of the symbol is: annotation, explanation of the meanings of the values in the table. ^a, b^ Values with different lowercase letters within the same row indicate significant differences (*P* < 0.05).

#### 3.1.3 Effect of age at calving season on milk quality

The data suggests the following results (Table 5). The calving season of Simmental cattle per parity had a significant effect on their milk production performance. Among the different seasons, Simmental cattle calving in winter exhibit higher average daily milk yield, while those calving in spring show significantly longer lactation days than other seasons (*P* < 0.05). However, no significant differences were observed between the seasons in terms of milk fat content and milk protein content, indicating relative stability in these two parameters. Considering milk yield and lactation days, Simmental cattle calving in winter might possess superior milk production performance.

**TABLE 5:** Effect of calving season on milk production performance of Simmental cattle.

Calving season	Average daily milk yield (kg))	Milk fat content (%)	Milk protein content (%)	Lactation days (d)
Spring	17.27 ± 2.71	3.93 ± 0.07	3.35 ± 0.03	372.05 ± 113.73^a^
Summer	18.53 ± 2.61	3.94 ± 0.07	3.36 ± 0.04	337.12 ± 76.87^ab^
Autumn	18.03 ± 3.06	3.96 ± 0.08	3.36 ± 0.03	317.82 ± 72.20^b^
Winter	19.15 ± 5.93	3.95 ± 0.09	3.36 ± 0.04	321.29 ± 78.97^b^

^*^ The values represent the mean ± standard deviation (SD), the meaning of the symbol is: annotation, explanation of the meanings of the values in the table. ^a, b^ Values with different lowercase letters within the same row indicate significant differences (*P* < 0.05).

### 3.2 Statistics and quality assessment of GBS-seq sequencing data

Based on the analysis of the provided GBS sequencing data statistics table, it was found that a total of 115 samples yielded 167.36 gigabases (Gb) of raw data, with an average of 0.91 Gb per sample. After quality control, the high-quality clean data amounted to 158.51 Gb, averaging 0.87 Gb per sample. The GC distribution was normal. Overall, the data met the expected range, indicating good sequencing data quality suitable for subsequent bioinformatics analysis and data mining tasks.

### 3.3 Reference genome matching and SNP detection

Upon aligning the filtered high-quality data to the reference genome of cattle, each sample exhibited an average of 5,987,693 reads aligned to the reference genome, with alignment rates exceeding 93.47%. The average sequencing depth was 8.24, and the Coverage of at least 4X per sample averaged around 2.95% (Supplementary Material S1). The results indicate that each sample meets the requirements for resequencing analysis, demonstrating good similarity to the reference genome, satisfactory coverage depth, and breadth. Subsequent population SNP detection on the samples revealed a total of 3,650,527 SNPs, of which 257,436 SNPs passed the filtering criteria for further analysis.

### 3.4 Principal components analysis and kinship analysis

**FIGURE 1:**
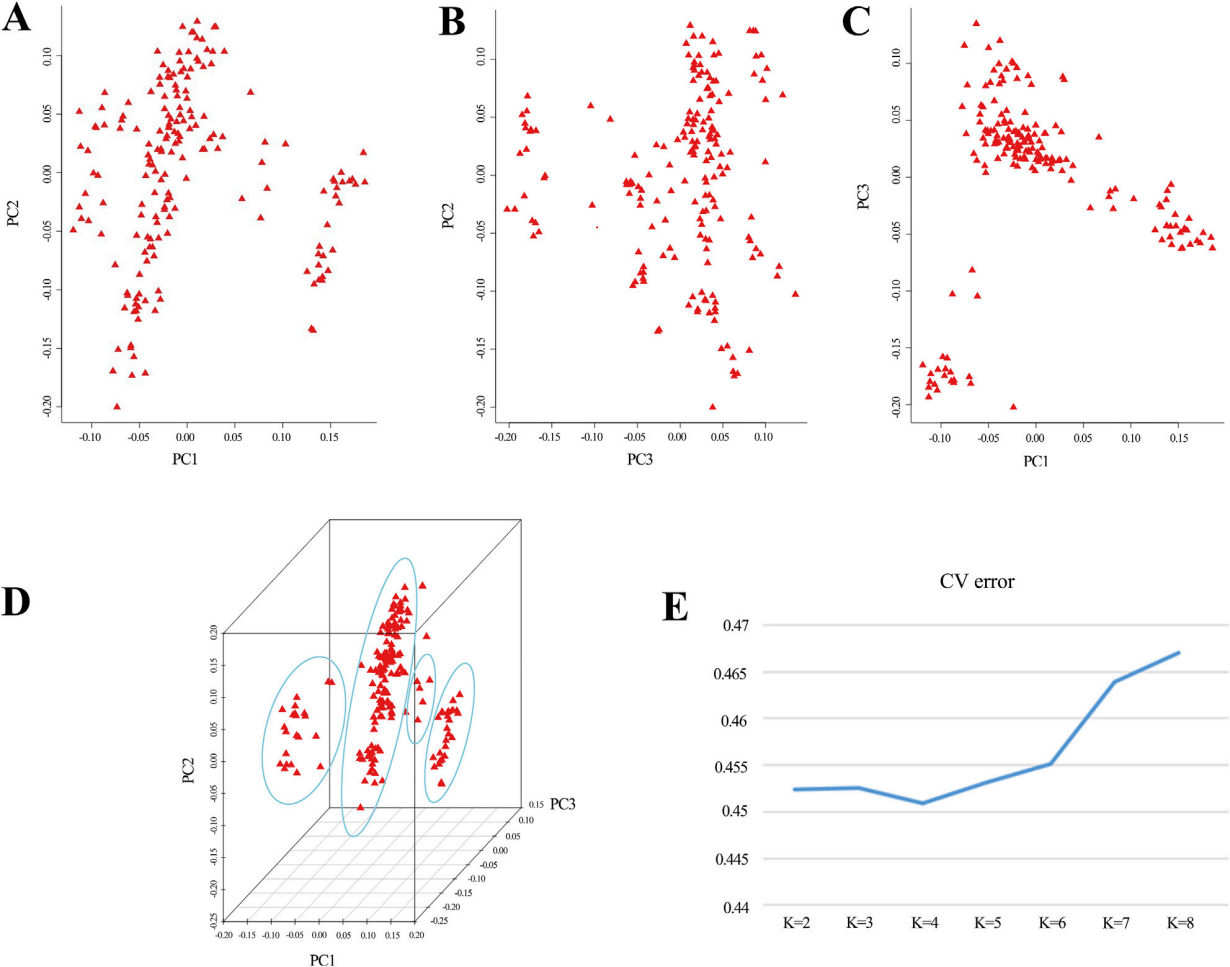
Principal component analysis reveals population structure. **(A–D)** The coordinates in the graph represent principal component 1, principal component 2, and principal component 3. Greater distances between samples indicate larger genetic differences. Ideally, individuals with similar genetic backgrounds cluster together in the graph. **(E)** A line graph depicts the CV error of Admixture.

Based on the outcomes derived from GCTA-1.24.2 software, eigenvectors and eigenvalues of the experimental population were computed, and a PCA distribution plot was generated using R software (Figures 1A–D). The analysis revealed clear population stratification, with 4 distinct subgroups observed. To control for potential confounding effects caused by population structure in the GWAS, the top three principal components were incorporated as covariates in the mixed linear model (MLM) for trait association analysis. Additionally, employing admixture-1.23 for the assessment of population genetic structure, the line plot (Figure 1E) demonstrated that the optimal K value, determined by the lowest CV error, was 4. The results from both analytical methods converge, indicating that the experimental population is best divided into 4 subgroups. Consequently, the 4 principal components would be integrated as covariates in the subsequent analyses.

### 3.5 GWAS analysis

**FIGURE 2:**
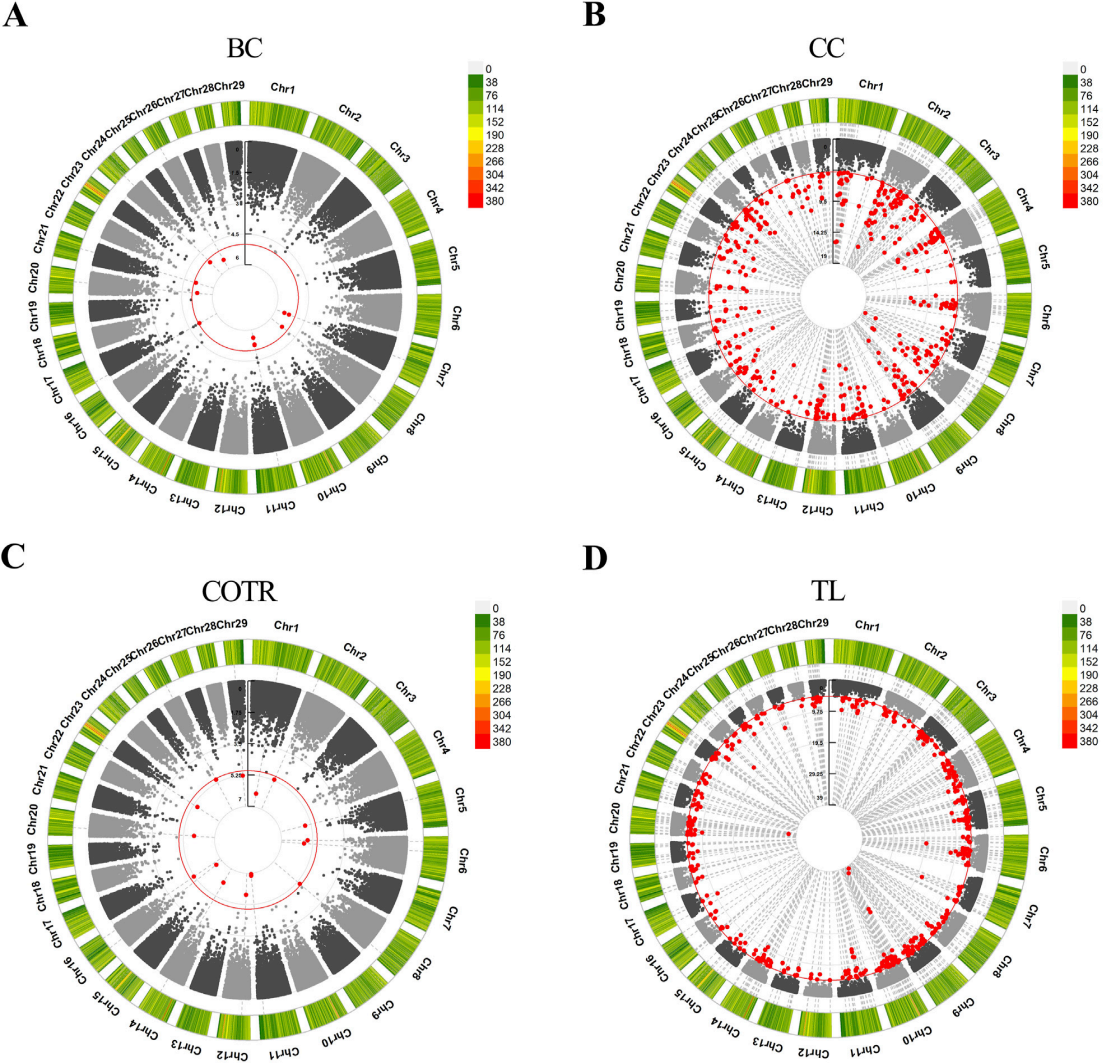
Manhattan plots were generated to depict the results of GWAS for four traits in Simmental cattle. **(A)** BC, body circumference. **(B)** CC, cannon circumference. **(C)** COTR, circumference over tail root. **(D)** TL, tail length. The outer circle represents the SNP-density plot, while the inner circle represents the circular Manhattan plot. The red circle is the threshold for the Bonferroni level of significance (*P* < 1 × 10^−5^), and the red dots within the circle represent significant SNP loci.

In the study, GWAS analysis was performed, and Manhattan plots were generated for each trait. 1,166 significant SNPs were identified at a significance threshold of P < 1 × 10^−5^. Subsequently, GWAS was conducted on four traits of 115 Simmental cattle, resulting in the annotation of a total of 584 candidate genes (Table 6, Supplementary Material S2; Supplementary Material S3). For the trait related to body size in Simmental cattle, regarding BC, 11 associated SNPs were identified, and 4 genes were annotated: *SH3RF2, RBM27, DCHS2*, and *PIK3AP1* (Figure 2A). Regarding the CC, significant SNP loci were observed distributed on each chromosome, with 253 candidate genes annotated. The top five candidate genes with the smallest *P*-values are *CAMK4, TMEM232, ADAMTS1, KCNJ3*, and *LOC107132327* (Figure 2B). GWAS results for the tail phenotype of Simmental cattle showed 18 significant SNP loci distributed across 13 chromosomes for COTR, annotating 9 candidate genes, including *IL1RAP, LOC112447424, FMNL3, LOC112449048, KIF26B, SWT1, LOC530348, LOC104968627,* and *RBMX2* (Figure 2C). For TL, 613 SNPs were identified, annotating 318 candidate genes. The top five genes with the smallest *p*-values are *THBS2, PKD2, CLINT1, LOC112449669*, and *FAM19A2 (* Figure 2D).

**TABLE 6:** Candidate genes corresponding to significant SNPs for target traits.

Trait	Candidate gene	Chromosome	Start	End
BC	*SH3RF2*	5	57144278	57164278
	*RBM27*	5	57454435	57474435
	*DCHS2*	6	3286880	3306880
	*PIK3AP1*	24	17961684	17981684
CC^a^	*ADAMTS1*	1	9591029	9611029
	*ROBO2*	1	24664491	24684491
	*ROBO1*	1	26851398	26871398
	*CADM2*	1	33163042	33183042
	*LOC112447291*	1	36387708	36407708
	*ST3GAL6*	1	43027751	43047751
	*CCDC141*	2	17969309	17989309
	*CCDC141*	2	18000265	18020265
	*…*	…	…	…
COTR	*IL1RAP*	1	76613094	76633094
	*LOC112447424*	1	76613094	76633094
	*FMNL3*	5	30169912	30189912
	*LOC112449048*	12	24551626	24571626
	*KIF26B*	16	31762721	31782721
	*SWT1*	16	66185786	66205786
	*LOC530348*	20	24219288	24239288
	*LOC104968627*	29	14368127	14388127
	*RBMX2*	29	14368127	14388127
TL	*EPHA3*	1	37513837	37533837
	*TRNAC-GCA-5*	1	61244099	61264099
	*CLDN1*	1	76961792	76981792
	*LOC104970909*	1	76961792	76981792
	*LPP*	1	78545396	78565396
	*B3GALT1*	2	28844558	28864558
	…	…	…	…

^a^ The complete table content of candidate genes for body circumference (BC), cannon circumference (CC), circumference over tail root (COTR), and tail length (TL) can be found in Supplementary Material S3 for reference.

### 3.6 Functional analysis of genes in regions related to target traits

**FIGURE 3:**
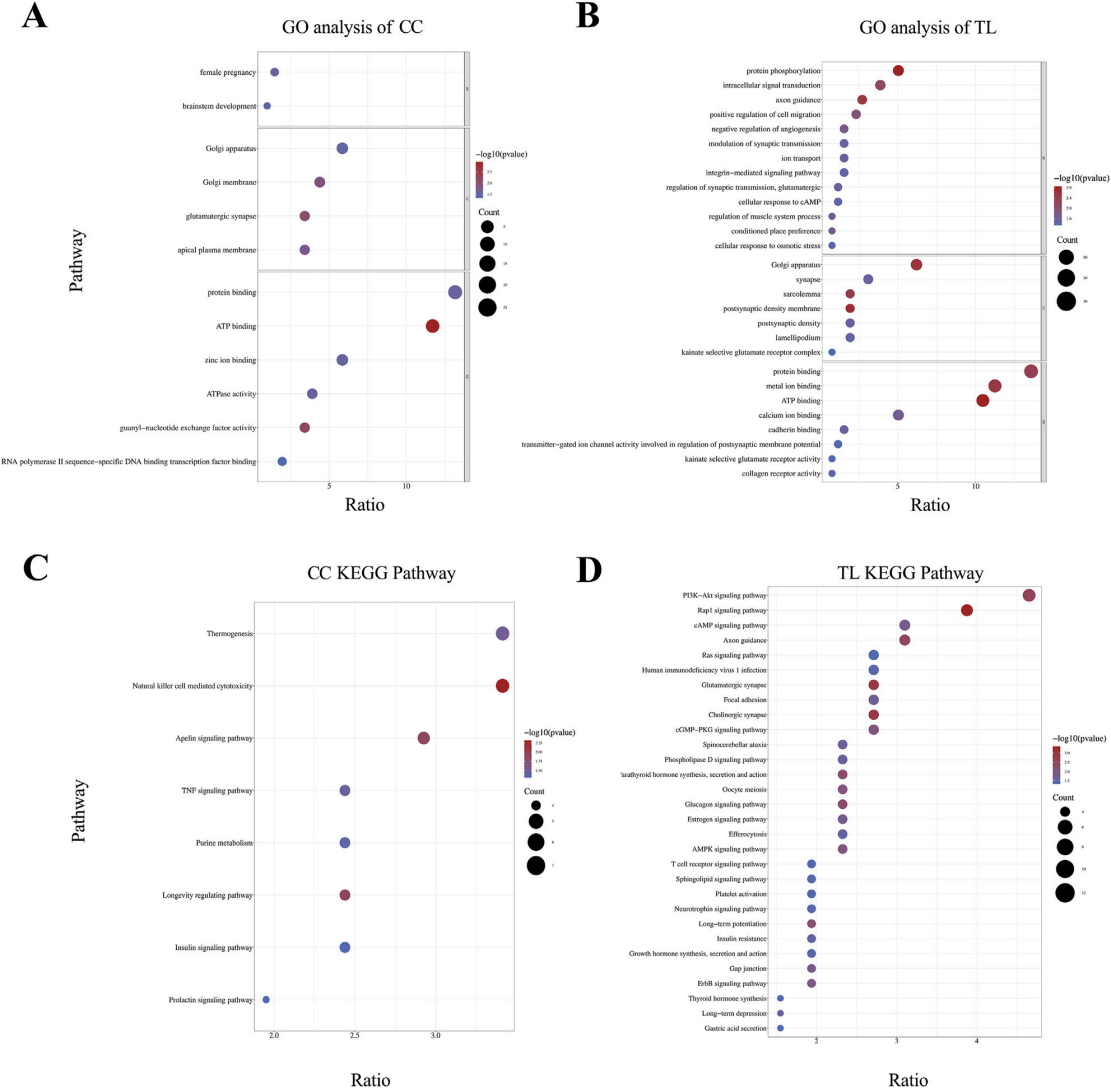
Enrichment analysis results of significant SNP candidate genes. **(A, B)** GO analysis was conducted on candidate genes associated with CC and TL traits, with significant enrichment terms visualized in the BP, CC, and MF categories. **(C, D)** KEGG pathway enrichment analysis of CC and TL trait.

Enrichment analysis revealed significant pathway enrichment for CC and TL traits. Figure 3 illustrates the results of the enrichment analysis. GO functional annotation analysis of the candidate genes for CC and TL traits identified multiple enriched terms across Biological Process (BP), Cellular Component (CC), and Molecular Function (MF) categories (Supplementary Material S4). The cannon circumference analysis revealed 12 annotated GO terms, with 2 terms significantly enriched in BP, 4 terms in CC, and 6 terms in MF (Figure 3A). The most represented terms in each category were “Female Pregnancy (GO:0007565),” “Golgi Apparatus (GO:0005794),” and “Protein Binding (GO:0005515),” respectively. The TL analysis identified 28 annotated GO terms, with 13 terms significantly enriched in BP, 7 terms in CC, and 8 terms in MF (Figure 3B). The top enriched terms were “Protein Phosphorylation” for BP, “Golgi Apparatus (GO:0005794)” for CC, and “Protein Binding (GO:0005515)” for MF. KEGG pathway enrichment analysis identified significant pathways for CC and TL traits (Supplementary Material S4).In the KEGG results for CC, the “Natural Killer Cell Mediated Cytotoxicity” pathway had the smallest *P*-value and highest ratio (Figure 3C). For TL-related KEGG pathways, the “PI3K-Akt signaling pathway” had the highest ratio, while the “Rap1 signaling pathway” had the smallest *P*-value (Figure 3D).

## 4 Discussion

The study unveiled the intricate interplay between AFC, parity, and milk production traits in Simmental cattle through statistical analysis, and a series of candidate genes were obtained using GWAS to dissect the different traits in cattle. By revealing the factors affecting milk yield and composition, as well as candidate genes influencing different traits in cattle, these findings offer practical insights for optimizing breeding strategies to improve milk production efficiency and quality of this breed. These insights are instrumental for farmers seeking to maximize production efficiency and economic returns from Simmental cattle.

### 4.1 Factors influencing milk production performance

As a dual-purpose breed for meat and milk, investigating milk production in Simmental cattle is meaningful and necessary. The parity of cows was identified as a determining factor influencing prepartum lactation performance (Fuerst-Waltl et al., 2004; González-Recio et al., 2012). The statistical analysis of phenotypic data revealed significant variations in udder depth and teat length among cows of different parities (*P* < 0.05), with the maximum udder volume observed in the third litter size, corresponding to the peak milk yield and lactation days. This is consistent with findings by Koc, Lee, Marumo, et al., demonstrating an increase in milk yield with parity, peaking at the third parity (Lee and Kim, 2006; Koç, 2011; Marumo et al., 2022). However, contrary to observations by Ehrlic and Dematawewa of longer lactation periods in primiparous cows (Ehrlich, 2013; Dematawewa et al., 2007), the study found that cows in their third parity had the longest lactation days. Analysis suggests that differences in lactation periods observed among studies might stem from variations in management practices, conditions, and overall herd management strategies. Additionally, unlike high-persistence primiparous cows, lower-persistence cows are less likely to experience negative energy balance post-calving, which does not affect subsequent estrus (Reksen et al., 1999). Further investigation into the specific factors influencing lactation periods in primiparous and multiparous cows could provide valuable insights for optimizing lactation management practices to maximize milk production efficiency.

The AFC is pivotal in cattle, as it signifies the non-productive period and serves as a crucial determinant of first lactation milk yield. Consequently, it is regarded as a vital predictor of subsequent milk production (Twomey and Cromie, 2023; Van Eetvelde et al., 2017; Van Eetvelde et al., 2020). Additionally, it correlates with more efficient lactation in the herd. Statistical results indicated optimal lactation performance in Simmental cattle at 3 years of age, contrasting with the findings of Kusaka et al. (2023) regarding Holstein cows calving at 22.5 months, likely due to the larger AFC in Simmental cattle, owing to their dual-purpose nature. Cows with smaller AFC tend to have higher survival rates. However, cows with larger AFC exhibit better economic performance than expected due to their higher milk production rates (Van Pelt et al., 2016). In larger cattle populations, a lower AFC has been documented, as reported by the United States Department of Agriculture (McCluskey, 2002). Individuals within larger herds exhibit enhanced reproductive performance metrics such as conception and pregnancy rates (Jago and Berry, 2011; Froidmont et al., 2013). This phenomenon might stem from heightened attention to husbandry practices in larger cattle operations, underscoring the importance of optimizing lactation management measures to ensure optimal milk production efficiency throughout the cow’s lifecycle.

Research on the impact of calving season on milk production performance has yielded inconsistent results across different cattle populations (Van Eetvelde et al., 2020). In the present study, milk production peaked during the winter season, followed by summer, a pattern consistent with findings by Maciuc (2009), Nor et al. (2013). However, this contrasts with the research of E. Froidmont et al. on Holstein cows, where summer recorded the highest milk yield (Froidmont et al., 2013). This disparity might arise from local climate conditions and calf-rearing practices. Additionally, calving season influences milk production performance, with reports indicating higher milk yield during the first lactation period for cows born in summer (Van Eetvelde et al., 2017; Soberon et al., 2012). Regarding milk protein composition, our findings align with those of G. Bufano et al., suggesting that calving season has no significant impact on compositional indices (Bufano et al., 2006).

In summary, the study provides valuable insights into the complex interactions among AFC, parity, and milk production characteristics in Simmental cattle. Understanding the factors influencing milk yield and composition dynamics enables breeders to implement targeted management practices, optimizing milk production efficiency and quality in Simmental cattle. These conclusions guide the optimization of management practices to maximize milk production efficiency and quality in Simmental cattle.

### 4.2 Body size traits and genetic associations in simmental cattle

Against the backdrop of continuous advancements in modern breeding techniques, pastoral management is increasingly transitioning towards modernization and scientificization. Breeders regard body condition scoring as playing a pivotal role in dairy cow husbandry (Oded, 1997). Body size parameters are critical for assessing the overall growth, development, and conformation of the cattle, which are essential for evaluating their suitability for breeding and production purposes. Anal-genital distance is a significant measure for understanding reproductive health and development, providing insights into sexual maturity, potential fertility issues, and identifying congenital abnormalities affecting reproductive efficiency. Linear evaluation indexes involve scoring various traits on a linear scale, including udder depth and teat placement. These scores allow for an objective assessment of physical traits, ensuring that desirable traits are passed on to future generations, thereby enhancing the overall quality of the herd. Apart from being utilized to assess the body weight of dairy cows, body measurement data also provide crucial information regarding slaughter rates, production performance, and overall health status (Li et al., 2022; Cominotte et al., 2020; Yao et al., 2022; Rezagholivand et al., 2021; Imaz et al., 2020). Through the evaluation and monitoring of dairy cattle’s physical characteristics, herdsmen could gain better insights into the growth and development of the cattle, thereby adjusting husbandry management measures accordingly. The traits of body measurements are typically quantitative traits controlled by multiple genes and their interactions, and multiple SNP in various genes have been demonstrated to be associated with body measurement data and meat quality traits (Liu et al., 2014; Abd El-Hack et al., 2018). Therefore, studying the SNP and candidate genes associated with these traits could provide an in-depth understanding of the genetic basis underlying body measurement traits. The study contributes to elucidating the genetic regulatory mechanisms of cattle conformation and meat quality traits (Tong et al., 2017; Lin et al., 2022).

Regrettably, despite the extensive investigation of individual Simmental cattle within the herd, the relatively small sample size and relatively consistent body weights among samples have somewhat restricted the statistical power to identify significant associations for body weight traits. Consequently, we did not observe a sufficient number of significant SNP, thus failing to yield significant results. This limitation suggests that future research on cattle population body weight would require larger sample sizes to enable more in-depth exploration. In the sequencing results for the BC trait, our analysis identified *SH3RF2* as a notable candidate gene. The *SH3RF2* gene is a member of the *SH3RF* protein family, which is distinguished by the presence of a single ring finger domain and multiple SH3 domains. Research has indicated that *SH3RF2* possesses the capability to inhibit apoptosis, as well as to promote cell migration and proliferation (Wilhelm et al., 2012; Kim et al., 2014). This gene has been identified in cattle studies for its influence on growth and testicular size in model animals and is one of the genes associated with double muscling in the Blonde d'Aquitane breed (Utsunomiya et al., 2014; Boitard and Rocha, 2013; Carreño et al., 2019). Additionally, research in chickens has positioned *SH3RF2* on a quantitative trait locus related to weight, demonstrating its effect on chicken weight (Jing et al., 2020; Rubin et al., 2010). These findings underscore the significance of the *SH3RF2* gene in regulating growth across different species. Further analysis suggests that this might be attributed to the correlation between BC size and the capacity of the cattle’s digestive system, thereby impacting growth functionality and ultimately weight and meat production. Similarly, the candidate gene *DCHS2* is associated with reproductive performance in pigs, and interestingly, significant correlations with weight have been observed in humans as well (Gu et al., 2017; Chang Wu et al., 2022). Hence, this cross-species evidence supports the hypothesis that *DCHS2* may similarly influence production traits in cattle, particularly those related to growth and weight. However, for other candidate genes, there remains a lack of relevant research in production aspects.

We identified several candidate genes significantly associated with the CC trait in Simmental cattle, providing new insights into the genetic architecture underlying body size traits. Among these genes, *ADAMTS1* showed notable relevance due to its previously reported expression in cattle leg muscles, promoting muscle stem cell activation (Du et al., 2017). Additionally, ADAMTS1 plays a role in milk fat percentage and various reproductive processes, such as follicle development, parturition, and spermatogenesis (Xia et al., 2021; Madan et al., 2003; Hernández-Delgado et al., 2023; McArthur et al., 2000; Mishra et al., 2013; Gurupriya et al., 2018; González-Barrio et al., 2021; Zhou et al., 2022). These findings suggest that *ADAMTS1* may influence both production and reproductive capabilities, further supporting its potential role in CC traits. Furthermore, the candidate gene *CAMK4* has been reported to play a significant role in cell fate and reproductive cell development, with enrichment in the oxytocin pathway. As an upstream regulator of Nitric oxide synthase, *CAMK4* could induce the production of NO in ovarian follicles, thereby impacting steroid hormone generation and follicle development (Tiwari et al., 2017; Nath and Maitra, 2019). It could also regulate lipid metabolism by enhancing insulin sensitivity (Li L. et al., 2020; Lee et al., 2014; Qin et al., 2024). Although there is currently no direct evidence linking this gene to physiological activities in cattle, considering its significant association with CC traits and potential mechanisms in regulating lipid metabolism, we hypothesize that the *CAMK4* gene might play a role in the growth, development, and metabolic regulation of cattle, which needs to be validated in subsequent experiments. Despite not having the smallest *P*-value, the candidate gene *PPARGC1A* might also be one of the important candidate genes. Previous studies have indicated that *PPARGC1A* plays a significant role in various biological processes, primarily through its regulation of mitochondrial production and energy metabolism. Numerous investigations have demonstrated that *PPARGC1A* influences both skeletal muscle and lipid metabolism by modulating glycolytic pathways and tricarboxylic acid (TCA) cycling (Ventura-Clapier et al., 2008; Dominy and Puigserver, 2013; Ma et al., 2022). Additionally, the research conducted by Manting Ma et al. further elucidates that *PPARGC1A* facilitates the conversion of fast muscle fibers to slow muscle fibers during chicken skeletal muscle development, suggesting that it may serve as a key candidate gene for enhancing chicken quality. The *PPARGC1A* gene has been reported to be significantly associated with milk yield and milk composition in dairy cows, as well as having a significant impact on calving interval and calving-to-conception interval (Komisarek and Walendowska, 2012; Weikard et al., 2005; Khatib et al., 2007; Pasandideh et al., 2015). Its potential involvement in CC traits highlights the complex genetic mechanisms influencing body size and production traits in cattle populations.

Among the candidate genes enriched in the GO analysis related to CC traits, *PRL, PRP6,* and *CORIN* were overrepresented in the pathway of female pregnancy (GO:0007565). This finding reflects the impact of reproductive status on body size during the developmental process in female animals. *PRL* is a significant factor in mammary gland development and lactation, further indicating the connection between CC traits and production capacity (Bole-Feysot et al., 1998; Ling et al., 2003). Additionally, we identified pathways related to organelle structure and function, such as the “Golgi apparatus” and “Golgi membrane” (GO:0000139) pathways. These pathways are associated with intracellular substance transport and secretion, and disruptions in these pathways may lead to metabolic imbalances, further influencing growth and development. This suggests a close relationship between CC traits and the regulation of animal body size. Interestingly, in the KEGG enrichment results, we noted the presence of the Longevity regulating pathway. While the specific function of this pathway in cattle remains unclear, it suggests a close association between CC traits and cattle growth, health, and longevity. Further research would help reveal the mechanistic role of this pathway in the biological processes of cattle and its impact on herd health and production performance.

### 4.3 Tail traits and production/metabolic traits in simmental cattle

The study observed significant differences in tail length among dairy cows with varying body weights. This observation prompted an exploration into the potential association between tail length and the metabolic status of dairy cows. The researchers hypothesized that these differences might stem from variations in metabolic activity within the cows, thereby influencing both body weight and the growth and development of their tails.

The tail serves as a crucial organ in the physiological and reproductive activities of cows (Alam et al., 2010; Salib and Farghali, 2016). However, due to its structural and physiological characteristics, coupled with the significant increase in metabolic activity during lactation, the tail is susceptible to various diseases such as dermatitis, injuries, necrosis, fractures, paralysis, and dislocations (Nuss and Feist, 2011). Among these, tail tip necrosis is reported as one of the most common diseases affecting the tails of beef and dairy cows. It initially presents with swelling at the tail tip, followed by inflammation within approximately a week. In advanced stages, tail tip necrosis might lead to muscular and articular, even pulmonary, purulent infiltration, resulting in substantial economic losses (Ural et al., 2007; Drolia et al., 1991). The underlying causes of these lesions might be attributed to reduced blood supply at the distal end of the tail and blunt trauma at proximal sites. Additionally, tail gangrene appears prevalent among dairy cows, likely caused by factors such as Corynebacterium bovis infection, fatty acid deficiency, and microfilaria infestation (George et al., 1970). Deg Nala disease, induced by fungal contamination in feed, results in necrosis and gangrene in the tails and legs of cattle (Irfan and Maqbool, 1986; Maqbool et al., 1998). The mycotoxins produced by fungal infestation of straw might cause vasoconstriction, exacerbating the lesions. Furthermore, tail rot is common in northern Australian cattle herds, possibly due to tail dislocation, fractures, or other injuries interrupting blood supply to the remaining portion of the tail below the wound. Moreover, metabolic abnormalities might also affect other organs and tissues, such as the liver and kidneys, thereby impacting overall health status.

In the sequencing results, several candidate genes associated with the COTR trait were identified. *KIF26B* is a member of the kinesin 11 superfamily, and previous studies have demonstrated its significant role in kidney development (Uchiyama et al., 2010). Research conducted in mice indicates that *KIF26B* is involved in embryogenesis, particularly in the development of limbs, facial structures, and body segments (Marikawa et al., 2004). Furthermore, *KIF26B* has been shown to regulate osteogenesis and chondrogenesis, crucial processes in tail tissue development (Pickering et al., 2018; Yan et al., 2022). As a key regulator of these processes, *KIF26B* might influence the bone structure and cartilage formation of bovine tails, suggesting its importance in determining tail length, pending further validation. Additionally, another candidate gene, *IL1RAP*, has been implicated in mediating inflammatory mediators (Yu et al., 2020). Inflammation is often intertwined with metabolic activities, particularly heightened during lactation in cows. *IL1R*AP might modulate the metabolic status of cows by regulating the expression of inflammatory mediators or signaling pathways, consequently affecting tail conditions and disease occurrence.

TL candidate gene *PKD2* is a member of the TRP (transient receptor potential) ion channel family and has different functions in multicellular organisms, including maintaining kidney function and regulating heart development (Zhang et al., 2023; Kim et al., 2009; Anyatonwu et al., 2007). Some studies have found that *PDK2* dysfunction often leads to male infertility and human reproductive defects, indicating that *PKD2* plays an important role in the reproductive system (Luciano and Dahl, 2014; Nakano et al., 2024). And *PKD2*has been determined to be associated with bone percentage, meat percentage, and meat-to-bone ratio in cattle (Niu et al., 2021; Abo-Ismail et al., 2014; Gutiérrez‐Gil et al., 2012). This indicates that the *PKD2* gene plays a role in regulating cattle body shape and meat production, and it also suggests that the TL trait might be related to meat production in cattle, which is consistent with the phenomenon that different tail lengths were found in cattle with different body weights and body shapes at the beginning of this study.

Enrichment in the calcium ion binding (GO:0005509) pathway was observed among the candidate genes associated with the TL trait, including *ITPR2*, *SLC8A1*, and *SLIT3*, all recognized for their involvement in bone formation (Zhang et al., 2015; Li et al., 2023; Hou et al., 2021; Li N. et al., 2020). This suggests their potential role in regulating bone formation processes in bovine tails, further implying their involvement in tail formation. Through participation in the calcium ion binding pathway, these genes likely play crucial regulatory roles in the development and shaping of bovine tail bones. This finding not only enhances understanding of the molecular mechanisms underlying tail formation but also provides valuable insights into the functions of these genes in other biological processes. Furthermore, the cellular response to the cAMP (GO:0071320) pathway has drawn attention, as it is closely associated with inflammation (Tavares et al., 2020), such as IL-10 production, leukocyte infiltration inhibition, and pro-inflammatory cytokine production (Serezani et al., 2008). The pathway might be one of the key pathways leading to bovine tail diseases. *AQP9, ITPR2*, and *PKD2*, among the candidate genes, were enriched in this pathway, further strengthening speculation regarding their potential significant roles in disease formation. The observation that the gene *ITPR2* is simultaneously enriched in both osteogenesis and inflammation pathways leads this study to consider it an essential gene, warranting further investigation into its specific functions and mechanisms in cattle tail formation and disease.

In the KEGG enrichment analysis, a large number of candidate genes were found to be enriched in pathways related to inflammatory responses, including the CAMP signaling pathway, CGMP-PKG signaling pathway, and AMPK signaling pathway (Lima et al., 2010; Peixoto et al., 2017). This deepens the understanding of tail diseases, suggesting a close association between tail health and metabolic inflammation. Further research would elucidate the specific roles of these enriched pathways in the occurrence and development of tail diseases. Through a comprehensive investigation into the functions and interactions of candidate genes within these pathways, the pathogenesis of tail diseases could be better understood, providing new possible targets and strategies for the prevention and treatment of bovine tail diseases in the future.

While our study identifies several candidate genes associated with tail traits, the underlying biological and functional mechanisms are not fully elucidated. Future research should focus on exploring the regulatory pathways and cellular processes linked to these genes to better understand their roles. Such insights could enhance the precision of molecular breeding strategies and improve the phenotypic selection of these traits in Simmental cattle. Additionally, future research should aim for larger sample sizes to facilitate robust sex-specific analyses and validate the findings from this preliminary study.

## 5 Conclusion

In conclusion, the study elucidates key findings regarding the genetic associations of milk production, body size traits, and tail length in Simmental cattle. Phenotypic data analysis revealed factors influencing milk yield in Simmental cattle, such as parity, AFC, and season. Additionally, GWAS on linear traits in Simmental cattle identified candidate genes potentially influencing morphological traits, including *SH3RF2* and *CAMK4*, and genes affecting production and reproductive performance, such as *DCHS2, PRL, PRP6, CORIN*, and *PPARGC1A*. Furthermore, GWAS analysis on tail identified various genes associated with tail characteristics, including *KIF26B, IL1RAP, ITPR2, SLC8A1, SLIT3, AQP9*, and *PKD2*. These genes might play crucial roles in determining tail length or the development of tail-related diseases in cattle. The study outcomes provide valuable insights for understanding genetic improvement and breeding in cattle, offering new molecular markers and targets for future research.

## Data Availability

The datasets presented in this study can be found in online repositories. The names of the repository/repositories and accession number(s) can be found in the article/Supplementary Material.
